# Acute Soft Head Syndrome: A Case Report in Kuwait and a Clinical Framework for Management

**DOI:** 10.7759/cureus.99232

**Published:** 2025-12-14

**Authors:** Noor Qali, Jafar Hayat, Refaa Al-Ajmi, Mariam M Salem, Shahad Al-Mubaraki

**Affiliations:** 1 Pediatrics, Al-Farwaniya Hospital, Kuwait City, KWT; 2 Pediatrics, Ministry of Health, Kuwait City, KWT; 3 Pediatrics, Kuwait Institute for Medical Specializations, Kuwait City, KWT

**Keywords:** acute soft head syndrome, ashs, clinical framework, sickle cell disease, subgaleal hematoma

## Abstract

Sickle cell disease (SCD) is a chronic, inherited hemoglobinopathy associated with multisystem complications, most commonly vaso-occlusive pain crises and stroke; however, rarer manifestations such as acute soft head syndrome (ASHS), characterized by painful scalp swellings from subgaleal hemorrhage, are often overlooked. We report the case of a 15-year-old Kuwaiti male with SCD who presented with a two-day history of headache, multiple scalp swellings, and low-grade fever, alongside a significant history of prior complications, including acute chest syndrome, stroke, splenectomy, and osteomyelitis. Laboratory investigations showed elevated inflammatory markers and leukocytosis, and neuroimaging revealed multiple subgaleal hemorrhages consistent with ASHS. The patient was managed conservatively with empirical antibiotics, hydration, and analgesia, with good clinical recovery. Based on this case and the limited existing literature, we propose a framework to aid early recognition and management of ASHS. This case highlights the importance of considering ASHS in the differential diagnosis of painful scalp swellings in patients with SCD, as timely recognition can support resolution with supportive care and prevent unnecessary interventions.

## Introduction

Sickle cell disease (SCD) is a chronic, inherited hemoglobinopathy affecting millions of people worldwide, with particularly high prevalence in sub-Saharan Africa, the Middle East, and parts of India [1]. The disorder results from the production of hemoglobin S, which causes red blood cells to assume a sickled shape under hypoxic or physiological stress, leading to vaso-occlusive crises (VOCs), hemolysis, and progressive multiorgan injury [2]. While common complications, including pain crises, stroke, and acute chest syndrome, are well-described, rarer manifestations remain underrecognized and may be overlooked in acute clinical settings [2,3].

One such rare and often misdiagnosed complication is acute soft head syndrome (ASHS), a clinically distinctive manifestation in which sudden, atraumatic scalp swelling develops due to subgaleal or subperiosteal hemorrhage, typically secondary to underlying calvarial bone infarction or osteonecrosis [4]. Although the exact pathophysiology is unclear, proposed contributing mechanisms include bone marrow hyperplasia, increased intracranial venous pressure, and vascular fragility [4,5]. ASHS presents a diagnostic challenge, particularly in children, as its clinical appearance may mimic infection, malignancy, or non-accidental injury [4]. Early recognition is essential because most cases are benign and self-limiting, resolving with supportive management and thereby avoiding unnecessary investigations or invasive procedures [4]. This report describes a pediatric case of ASHS in a patient with SCD, emphasizing the importance of clinician awareness of this rare entity. We also provide a brief review of the literature and outline a proposed framework to guide recognition and management.

## Case presentation

We report the case of a 15-year-old Kuwaiti male patient of Black ethnic origin. He has known SCD, β-thalassemia trait, and glucose-6-phosphate dehydrogenase (G6PD) deficiency. He was first diagnosed with SCD at six months of age following an episode of dactylitis. Since then, he has experienced multiple SCD-related complications, with approximately six hospital admissions per year for vaso-occlusive crises. Major events have included splenectomy at five years of age following recurrent splenic sequestration with hemolysis requiring several blood transfusions per year; acute chest syndrome with subsequent lung fibrosis at seven years of age; a cerebral infarct resulting in generalised tonic-clonic seizures, treated with antiepileptic medication for three months and exchange transfusion; and osteomyelitis with secondary osteonecrosis of the right hip and lumbar vertebrae at 14 years of age. He is also followed by endocrinology for short stature and receives growth hormone therapy, and by cardiology for a patent foramen ovale with a right-to-left shunt. Additional comorbidities include bilateral flat feet and major depressive disorder. His regular medications include hydroxyurea, penicillin, L-glutamine, deferasirox, and folic acid. Although these comorbidities are not known to have a direct association with ASHS, they are included here to provide a complete overview of the patient’s medical background.

He presented to the emergency department (ED) with a two-day history of headache, initially localized to the vertex, which woke him from sleep and was pulsatile in nature. The headache was worse on bending forward and was associated with a subjectively reported fever. It responded partially to simple analgesia, including ibuprofen. One day after the onset of the headache, he noticed four distinct swellings over the left side of the scalp (two frontal and two parietal), each approximately 2 cm in diameter. The swellings were tender to palpation, particularly when brushing or combing his hair. They subsided spontaneously one day after appearing, although the scalp remained diffusely tender. There was no history of head trauma.

**Figure 1:**
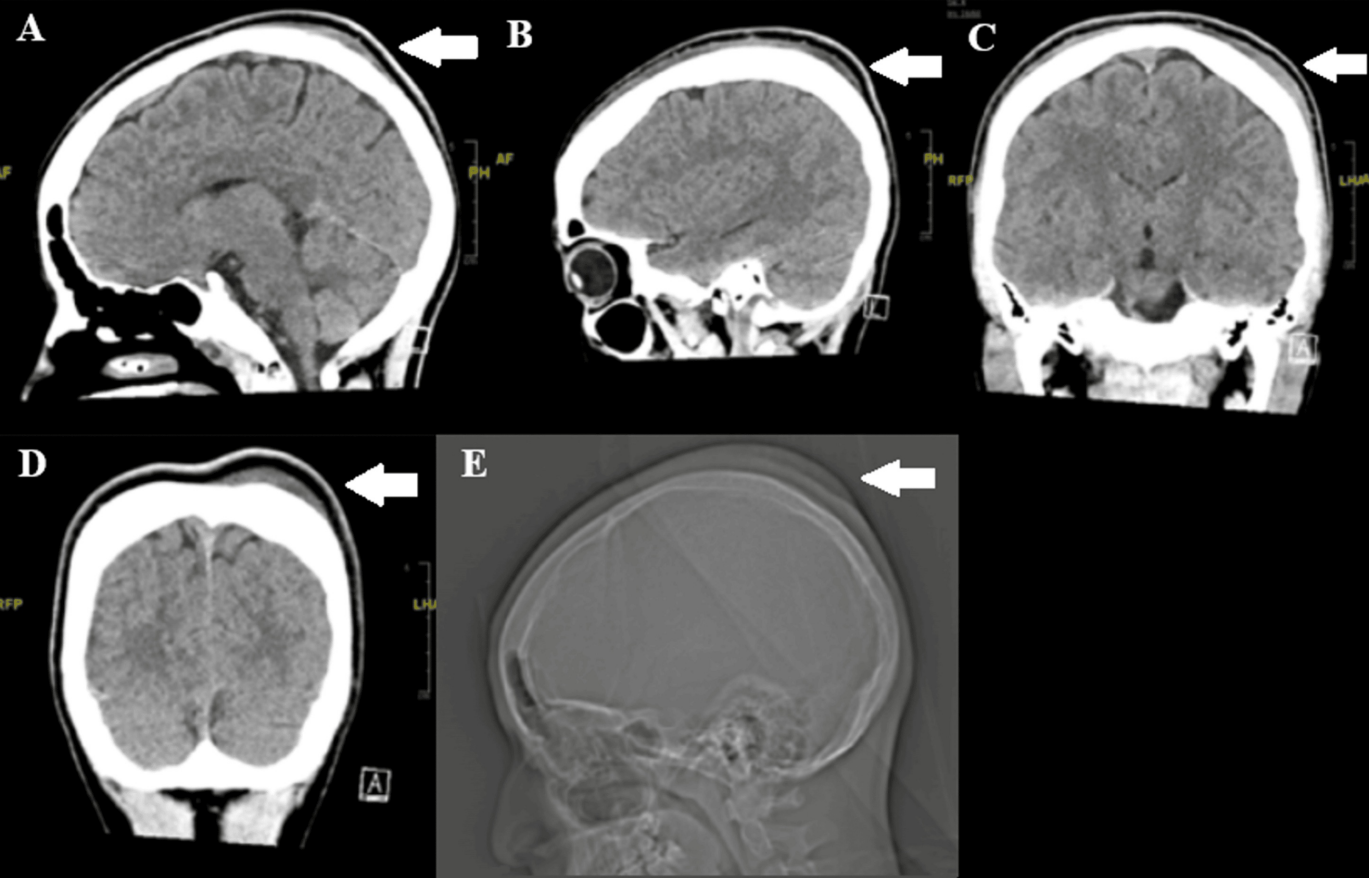
Computed tomography (CT) imaging of the patient (A) Sagittal noncontrast CT demonstrating a well-defined, homogenous, hypodense subgaleal hematoma over the left frontoparietal region, measuring approximately 16 mm at maximal thickness. (B) Additional sagittal slice showing the same hypodense collection with an overlying outward contour bulge of the scalp. (C) Coronal CT view illustrating the full transverse extent of the homogenous subgaleal fluid collection across the left frontoparietal region. (D) Lower coronal slice further delineating the low-attenuation subgaleal collection and associated scalp swelling. (E) CT scout image demonstrating the external contour prominence produced by the subgaleal hematoma The images above demonstrate CT and X-ray imaging of the patient, demonstrating a large subgaleal hematoma of the left frontoparietal region, measuring approximately 16 mm at maximum thickness, with an overlying contour bulge

On examination in the ED, the patient appeared clinically well. Physical examination was unremarkable apart from mild pallor. His temperature was 38.3°C, and his pulse rate was 116 beats per minute; blood pressure and oxygen saturation on room air were within normal limits. During this admission, he received supportive management with intravenous (IV) fluids at 1.5 times the maintenance rate and paracetamol and ibuprofen as required. In view of his post-splenectomy functional asplenia and concerns for osteomyelitis, the infectious diseases team recommended empirical IV vancomycin and aztreonam from day 3 of admission; IV meropenem was also added on day 3 alongside aztreonam. He remained afebrile after the first hospital day, and symptoms of pain gradually improved over several days. Neurosurgery was consulted and advised against needle aspiration of the scalp swellings. He was discharged on day 6 with a planned follow-up in the neurosurgery and hematology clinics. Laboratory investigations taken on admission day and day 3 of his stay, and CT imaging taken on day 1 of his stay, are summarized in Table 1 and Figure 1.

**Table 1:** Lab investigations of the patient Bloodwork was collected on days 1 and 3 of the patient's stay in the hospital. There is a mild drop in Hb as well as downtrending of acute inflammatory markers. Further investigations, including renal function tests, nasopharyngeal viral swabs, and urine and blood cultures, were unremarkable

Lab test	Day of admission	Day 1	Day 3	Reference range
Hemoglobin (g/L)	98	–	93	120-160 g/L
Reticulocyte count (×10⁹/L)	0.18 (3.96%)	–	–	0.02-0.08 (0.5-2.5%)
Lactate dehydrogenase (U/L)	428	–	–	140-280 U/L
C-reactive protein (mg/L)	169	–	94	<5 mg/L
Erythrocyte sedimentation rate (mm/hr)	20	–	–	<15 mm/hr
Procalcitonin (ng/mL)	0.13	–	0.05	<0.05 ng/mL

Follow-up and outcome

One week after discharge, the patient re-presented with a vaso-occlusive crisis involving the jaw, chest, and knees. He was admitted and managed with IV fluid hydration and analgesia as required. Admission blood tests (Table 2) showed markedly elevated inflammatory markers and a reduction in hemoglobin. On the second day of admission, he reported two tender frontoparietal scalp swellings associated with a band-like headache that worsened with sudden movement, as well as photophobia, phonophobia, and nausea.

**Table 2:** Relevant bloodwork collected of the patient throughout their stay Routine bloodwork was collected during days 1, 6, 8, 11, and 13 of the patient's stay in the hospital. There is a moderate drop in Hb, which improved after blood transfusion. Further investigations, including liver function tests, renal function tests, nasopharyngeal viral swabs, and urine and blood cultures, were unremarkable

Parameter	Day of admission	Day 1	Day 6	Day 8	Day 11	Day 13	Reference range
Hemoglobin (g/L)	88	73	76	98	92	–	120-160 g/L
Reticulocyte count (×10⁹/L / %)	0.1935 (4.49%)	–	–	–	–	–	0.02-0.08 (0.5-2.5%)
C-reactive protein (mg/L)	390	–	–	–	–	–	<5 mg/L
Erythrocyte sedimentation rate (mm/hr)	110	–	–	–	–	–	<15 mm/hr
Procalcitonin (ng/mL)	0.18	–	–	–	–	–	<0.05 ng/mL
G6PD level (U/g Hb)	8.76	–	–	–	–	–	7-20 U/g Hb*

**Figure 2:**
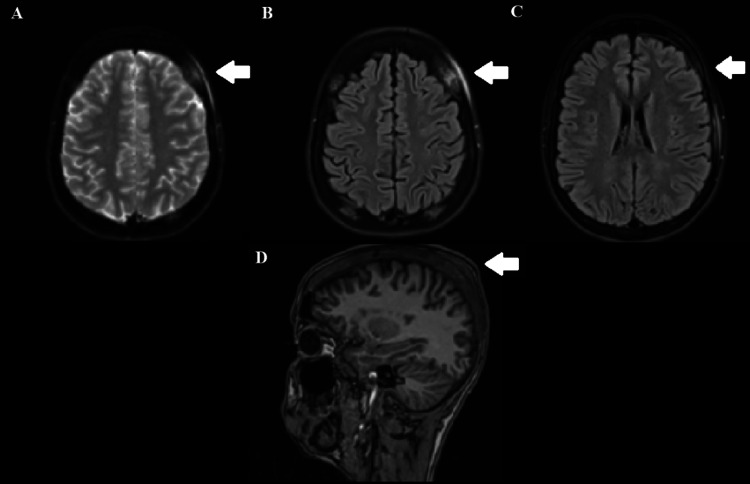
MRI sequences demonstrating calvarial and extracranial changes consistent with ASHS and possible bone infarction ASHS: acute soft head syndrome; FLAIR MRI: fluid-attenuated inversion recovery magnetic resonance imaging (A) Axial T2-weighted MRI showing diffuse expansion of the calvarial bones with patchy areas of abnormal marrow signal. (B) Axial FLAIR MRI demonstrating peripheral marrow enhancement and heterogeneous signal intensity, suggestive of early bone infarction or osteomyelitis. (C) Axial FLAIR MRI at a lower slice demonstrating small, noncontiguous subgaleal and epidural collections, more prominent at the frontoparietal region. (D) Sagittal T1-weighted MRI demonstrating marginal enhancement of the subgaleal collection and reactive changes in the overlying soft tissues. Major intracranial vessels appear unremarkable, with no evidence of territorial ischemia on MR angiography Magnetic resonance imaging (MRI) demonstrating diffuse expansion of skull calvarial bones with multiple bone marrow patches of abnormal signaling, predominantly peripheral enhancement, suggestive of potential bone infarction or osteomyelitis. Further noted a few small noncontiguous subgaleal and epidural collections, with the most significant at the frontoparietal region, revealing marginal enhancement, suggestive of tissue reaction or infective changes. Unremarkable MR angiography findings, with no evidence of ischemic insult to the major territories

Analgesia was optimized, including the use of parecoxib in addition to paracetamol and ibuprofen. The headache improved over the following two days; however, he developed fever on the fifth and sixth hospital days, and IV piperacillin-tazobactam was commenced because of concern for osteomyelitis. By day 6, the headache had again improved, and he remained apyrexial. On day 7, the headache reached maximal severity and became refractory to his usual analgesia, prompting an increase in the dose of parecoxib. MRI of the brain and skull (Figure 2) was reviewed with the infectious diseases team, who felt the findings were more consistent with bone infarction than osteomyelitis; antibiotics were therefore discontinued. By day 9, the headache was improving further. On day 10, he underwent an exchange transfusion for severe vaso-occlusive pain, with pre- and post-exchange hemoglobin levels shown in Table 3. Following exchange transfusion, the headache resolved completely, and he was discharged home on day 14.

**Table 3:** Serum electrophoresis of the patient prior to and post-exchange transfusion HbA: hemoglobin A; HbA2: hemoglobin A2; HbF: fetal hemoglobin; HbS: hemoglobin S

Hemoglobin type	HbA	HbA2	HbF	HbS
Pre-exchange transfusion %	8.7%	5.7%	8.4%	77.2%
Post-exchange transfusion %	56.9%	4%	3%	36.1%

At the outpatient neurosurgery review two weeks later, the patient remained clinically stable, with complete resolution of scalp tenderness and no recurrence of swellings. Repeat laboratory investigations showed normalization of hemoglobin and inflammatory markers, with no evidence of ongoing infection or hemolysis. Over subsequent routine hematology visits in the following months, he has had no further hospital admissions or episodes of scalp swelling or severe headache. His clinical course is therefore consistent with the benign, self-limiting nature of ASHS, and he continues under regular follow-up for chronic SCD management.

## Discussion

ASHS, also referred to as sickle cell cephalohematoma, is a rare but significant complication associated with SCD. It is characterized by acute scalp pain and swelling occurring in the absence of trauma [4,5]. Patients may present with diffuse or localized scalp swelling that is tender to palpation and commonly associated with a headache lasting hours to days [4]. Focal neurological deficits are typically absent. Several reports also describe the presence of low-grade fever at symptom onset [4,6-9]. Our patient presented with a parietotemporal headache, tender scalp swellings, and a documented low-grade fever at the time of evaluation in the emergency department.

The underlying pathophysiology of ASHS is not fully understood. The most widely accepted hypothesis links the condition to vaso-occlusive events leading to skull bone marrow infarction and subsequent tearing of small vessels [4]. Although bone infarction can occur in any bone, it is more commonly observed in the long bones, ribs, sternum, spine, and pelvis; the skull is less frequently affected [7]. Recurrent VOCs are also thought to contribute to the development of ASHS through multiple small infarcts that progressively weaken the structural integrity of bone and periosteal tissues. These changes may culminate in vessel wall necrosis and leakage of blood into the subgaleal or epidural spaces, producing the clinical findings seen in ASHS [8].

ASHS appears to occur predominantly in adolescent males with SCD [4,6,9-14], although adult cases have also been reported [15,16]. Bone infarction may also result in epidural hematoma rather than subgaleal collection; however, such cases typically present with headache in the absence of scalp swelling and therefore fall outside the current definition of ASHS [17,18]. Subgaleal hematoma with periorbital swelling in SCD has also been described [5].

The nonspecific presentation of ASHS can be challenging, as it overlaps with several important differential diagnoses, including nonaccidental injury, malignancy, infection, and bleeding disorders. Osteomyelitis is the most critical alternative diagnosis to exclude, given its higher incidence in SCD and its potential severity. Osteomyelitis often presents with unilateral swelling, persistent fever, markedly elevated inflammatory markers, and may be accompanied by positive blood cultures [19]. In our case, osteomyelitis was considered but ultimately excluded based on imaging findings. Both CT and MRI demonstrated multiple subgaleal and epidural collections without cortical destruction or periosteal reaction, features more consistent with bone infarction. Additionally, the lesions were noncontiguous, lacked significant enhancement, and preserved subcutaneous fat planes, further supporting a noninfectious etiology.

Diagnosis of ASHS relies on clinical suspicion and familiarity with this rare manifestation of SCD. Laboratory investigations may show leukocytosis, acute hemolysis, and elevated inflammatory markers. Imaging plays a central role, with MRI being the preferred modality when available. MRI findings typically include diffuse calvarial thickening and abnormalities in bone marrow signal intensity. Hyperintense T2-weighted or fluid-attenuated inversion recovery (FLAIR) sequences and heterogeneous T1-weighted signals are suggestive of marrow edema and infarction [20]. MRI additionally aids in excluding alternative diagnoses such as acute stroke or intracranial hemorrhage, further strengthening the diagnosis of ASHS [21]. CT imaging, while less sensitive for marrow pathology, remains a useful initial tool in the acute setting.

Most reported cases of ASHS resolve with conservative management, including treatment of vaso-occlusive crises with intravenous fluids and analgesia [6,9]. Exchange transfusion may be beneficial in selected cases and may help reduce the severity or recurrence of symptoms. In our patient, symptoms improved with supportive measures during both episodes, with additional symptom relief following exchange transfusion during the second presentation.

The literature does not clearly define the expected duration of symptom resolution, although one report documented complete MRI resolution at one-year follow-up [6]. The potential for recurrence remains uncertain; however, our patient experienced a second episode of ASHS approximately one week after initial recovery. Nonetheless, he responded well to conservative therapy on both occasions.

**Figure 3:**
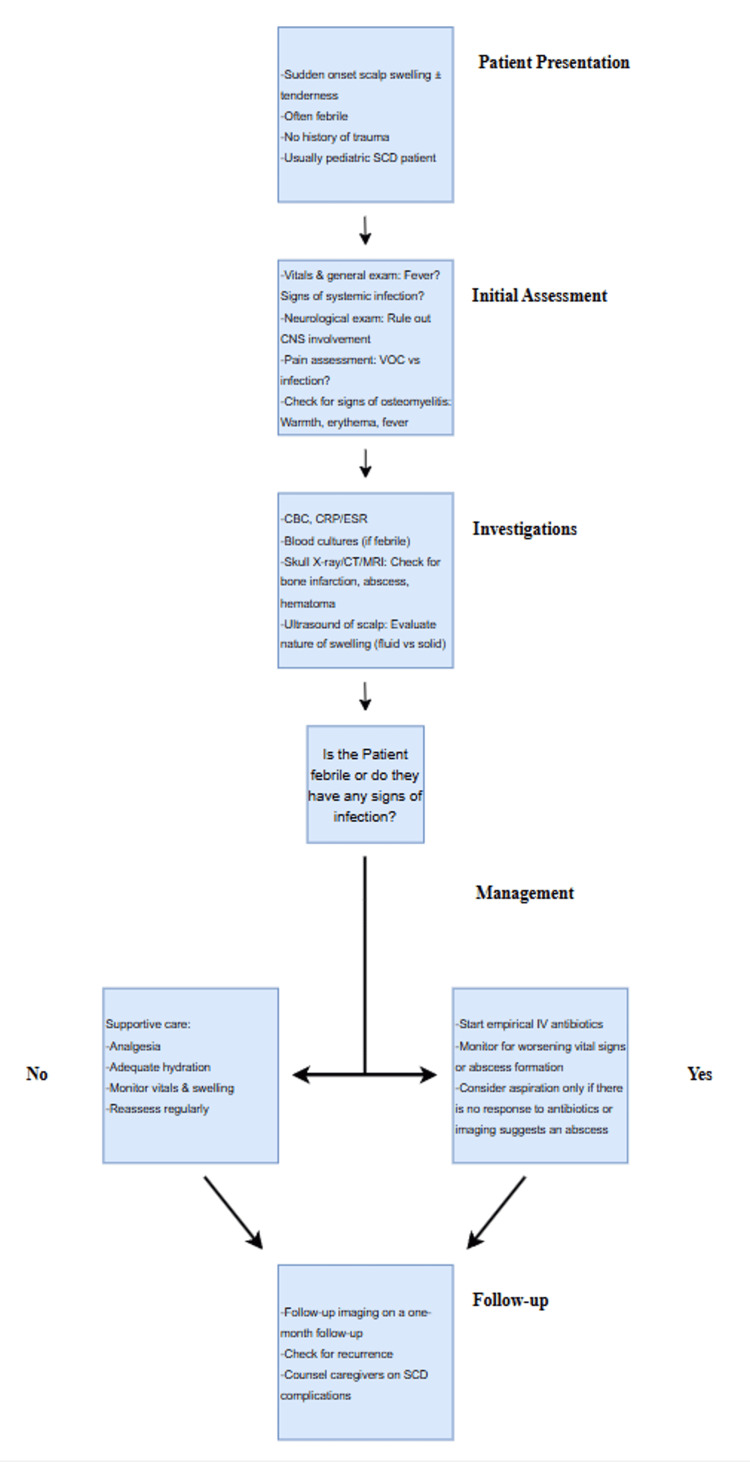
A proposed management framework for the presentation, investigations, management, and follow-up of ASHS ASHS: acute soft head syndrome

Follow-up imaging may be appropriate under neurosurgical guidance to monitor the resolution of ASHS, particularly in cases with atypical features or recurrent symptoms. Ongoing optimization of chronic SCD management is essential following an SCD-related complication, and reassessment of disease-modifying therapy or the potential need for regular exchange transfusions may be warranted to reduce future sickling events. A summary of our proposed framework for the presentation, clinical course, and management of ASHS, based on our case and existing literature, is presented in Figure 3.

## Conclusions

Our case highlights the importance of recognizing rare complications of common conditions such as SCD. Skull bone infarction with secondary subgaleal collection is an uncommon but important cause of acute headache and scalp swelling in this population. This complication is typically benign and self-limiting, with most cases managed conservatively through empirical antibiotics when clinically indicated, analgesia, and intravenous hydration. The clinical course is generally short and uncomplicated. Further research is needed to better define the pathophysiology of ASHS, its optimal management, and long-term follow-up strategies.
